# Health risk perception and exercise intention of college students: a moderated mediation model of health anxiety and lay theories of health

**DOI:** 10.3389/fpsyg.2024.1375073

**Published:** 2024-04-30

**Authors:** Kun Wang, Chen Liu, Xiao Yang, Yue Wang

**Affiliations:** ^1^Department of Physical Education, Zhengzhou University of Economics and Business, Zhengzhou, China; ^2^School of Education, Zhengzhou University, Zhengzhou, China

**Keywords:** health risk perception, health anxiety, lay theories of health, exercise intention, college students

## Abstract

**Background:**

Health risk perception is an important predictor of health-protective behaviors according to the health belief model. However, the underlying mechanism connecting health risk perception and exercise behaviors is not well understood. The current study investigates how health risk perception predicts college students' exercise intention in the post-pandemic era in China and analyzes the mediating effect of health anxiety and the moderating effect of lay theories of health.

**Materials and methods:**

This cross-sectional study adopted convenience sampling and recruited 767 students from a province in central China. The Health Risk Perception Scale, Health Anxiety Scale, Lay Theories of Health Scale, and the Chinese version of the Exercise Intention Scale were used to measure the levels of health risk perception, health anxiety, implicit health theory, and exercise intention, respectively.

**Results:**

The results of the moderated mediation model indicated that the health risk perception of college students significantly and positively affected exercise intention (β = 0.110, *t* = 2.852, *p* < 0.01). Meanwhile, the indirect effect of health anxiety on the relationship between health risk perception and exercise intention was significant. Furthermore, lay theories of health buffered the association between health anxiety and exercise intention, according to the moderated mediation analysis (β = 0.068, *t* = 2.067, *p* < 0.05). For college students holding incremental health theory, the influence of health anxiety on exercise intention was positively and statistically significant.

**Conclusion:**

The health risk perception of college students can lead to health anxiety, which can positively affect their exercise intention. In addition, lay theories of health can moderate the effect of health anxiety on exercise intention. The results have practical implications for developing effective, applicable, and scalable interventions to promote physical exercise by reducing the level of entity theory of health or increasing the level of incremental theory.

## 1 Introduction

During the COVID-19 pandemic, given the lack of effective antiviral drugs to control the pandemic and prolonged time expected for achieving herd immunity through vaccination, people largely adopted health preventive measures such as wearing masks and maintaining social distancing (Iorfa et al., 2020). The post-pandemic era is witnessing a growing awareness and recognition of the importance of improving one's physical fitness and disease resistance. It is well known that engaging in physical exercise is one of the most effective ways to improve physical fitness. Regular physical exercises can promote overall physical health (e.g., physiological functions such as vital capacity and strength, ideal body fat percentage, and ideal body mass index) (Da Silveira et al., 2020). However, according to the results of the Eighth National Survey on Students' Physical Condition and Health released by the Ministry of Education, China (2021), less than one-quarter of the participants received excellent and good ratings according to the body shape, physiological function, physical fitness, health status. Existing studies have highlighted that sedentary lifestyles and lack of physical activity are currently being recognized as social problems in the world and risk factors for individuals' health. In general, healthy development of teenagers' physiques is still not optimistic. Therefore, guiding college students to improve their engagement in physical exercise and develop exercise habits has become crucial for improving their physical fitness. From a theoretical standpoint, understanding the determinants of physical exercise can provide information for developing more effective interventions to promote behaviors that reduce health risks. According to the theory of planned behavior, intention directly influences behavior (Hou et al., 2022). Exercise intention can significantly and positively predict exercise behavior (Chen et al., 2022). This study explored the internal mechanism of the impact of health risk perception on physical exercise intention under the framework of the health belief model and implicit health theory in order to provide a reference for improving physical exercise intention and the status quo of physical exercise among college students.

### 1.1 Health risk perception and physical exercise

Risk perception has been commonly studied as a key psychological variable influencing health behavior. Health risk perception is used to describe people's attitudes and perceptions of objective health risks, involving diseases, technology, living habits, the environment, and many other fields, and it is one of the key driving factors of health-related behaviors (Lei et al., 2018; Ec et al., 2020). Many scholars applied health behavior change theories such as protection motivation theory (PMT); knowledge, attitude, and practice (KAP); and the Health Belief Model (HBM) to conduct studies on risk perception and coping behaviors. These studies provide a consensus that individuals' perception of health risks can primarily affect initiating or maintaining their positive health behaviors or avoiding negative behaviors (Kim, 2018). Previous research demonstrated that people's risk perception significantly predicts their responses to infectious disease pandemics; specifically, people who perceived a higher level of risk were more likely to engage in health-protective behaviors (Rudisill, 2013; Sheeran et al., 2014). Another study reported that there is a significant correlation between risk perception and self-reported preventive health behaviors (Dryhurst et al., 2020). Engaging in physical exercise is a widely endorsed approach for reducing health risks. It is well established that performing physical activities is one of the significant health protective behaviors to prevent and reduce many types of health problems. However, inconsistency exists in the findings of several studies. Evidence suggests that risk perception has a significantly positive effect on physical exercise intention (He et al., 2022). Health risk perception can primarily affect initiating or maintaining positive health behaviors (i.e., physical activity) among older adults and pregnant women (Stephan et al., 2011; Connolly et al., 2016). Another study contradicts these findings, demonstrating that the sense of control rather than risk perception has a significantly positive effect on the persistence of exercise behaviors (Xi and Wang, 2021). Currently, there is insufficient evidence to support the relationship between health risk perception and physical exercise intention, as well as the psychological mechanisms that underpin this relationship. Based on the above literature review, we regard exercise intention as a response to an individual's perception of health risks and a self-protective behavior adopted by an individual. Accordingly, we propose the hypothesis that health risk perception can positively predict college students' exercise intention (Hypothesis 1).

### 1.2 The intermediary role of health anxiety

It has long been recognized that risk perception is closely related to emotions (Sjöberg, 2007). On the one hand, the risk perception caused by negative emotions can not only decrease the state of an individual's mental health but also increase the individual's risk behavior level (Hu et al., 2013). On the other hand, risk perception can also affect emotions. A large number of studies have investigated individuals' emotional experience and its relationship with their risk perceptions during an epidemic outbreak. These results showed that there is a close relationship between individuals' risk perceptions and negative emotions. The risk information seeking and processing model (RISP) (Yang et al., 2014) proposes that, when individuals perceive a risk event, to ensure their own health and safety, they usually attempt to adopt preventive attitudes, beliefs, and behaviors. Individuals will also have negative emotions such as worry and anxiety during this process. Numerous studies claimed that, during the outbreak of COVID-19, there was a significantly positive correlation between risk perception and anxiety or depression (Li et al., 2020; Zhang and Wang, 2023). A recent meta-analysis study provided further evidence that risk perception is strongly associated with fear and moderately associated with anxiety (Zhao et al., 2023). An individual's worry about their physical and psychological health is broadly referred to as health anxiety and can range from mild concern to severe or persistent anxiety such as that found in Diagnostic and Statistical Manual of Mental Disorders (DSM-IV) hypochondriasis (Commons et al., 2016). Within reasonable bounds, health anxiety is common in society and is not considered a disease, but rather, it is a factor that contributes to protecting body integrity and health. It can prompt individuals to engage in health-promoting behaviors. As a negative emotion, health anxiety can be an important defense mechanism that can prompt people to react accordingly. For example, a cross-sectional study showed that adolescents with higher health anxiety engage more intensively in health-related social media use and health information-seeking behaviors or exhibit higher health consumption intention and health maintenance behaviors (Liang et al., 2023; Lokajova et al., 2023).

There is a mass of evidence showing that exercise can reduce anxiety and depression, whereas a lack of exercise is associated with higher levels of anxiety and depression (Kemp et al., 2021). The effect of negative emotions (especially in the field of health) on physical exercise behaviors is rarely mentioned. Although previous research indicated that health anxiety is associated with reassurance seeking, the relationship between health anxiety and wellness-related behaviors, such as engaging in physical activities, has not been adequately examined. Health anxiety can be potentially related to exercise dependence (Weinstein et al., 2015). More specifically, it was indicated that individuals who experience higher levels of anxiety symptoms are more likely to have the desire to exercise compared to those with lower levels of anxiety (Pugh and Hadjistavropoulos, 2011; Back et al., 2021). This finding implies that individuals who experience higher levels regard exercise as a strategy to cope with their anxiety. A study that found an association between health anxiety and the desire to exercise that adds some credence to this viewpoint (Gülhan et al., 2021). In addition, a cross-sectional study determined that health anxiety increases the tendency toward healthy lifestyle behaviors such as exercise, good nutrition, and positive interpersonal relationships. Therefore, in this study, we argue that health anxiety may play a particular role in the correlation between health risk perception and exercise intention. Specifically, this study hypothesizes that health risk perception affects exercise intention through the mediating role of health anxiety (Hypothesis 2).

### 1.3 The moderating effect of lay theories of health

One psychological mechanism that draws increasing attention among health psychologists in predicting health behaviors is people's lay theories of health (Dweck et al., 1995). The lay theories of health could be categorized as incremental and entity theories. Individuals with incremental theory tend to believe that their health levels can be improved through their own efforts, whereas those with entity theory believe that their health levels are more influenced by congenital factors and are hard to change (Zhang and Kou, 2022). For example, previous research demonstrated that individuals who tend to consider health as malleable (incremental) are more likely to engage in health behaviors than those who tend to consider health as unchangeable (entity) (Bunda and Busseri, 2019). A recent study showed that lay theories of health can predict the possibility of people participating in health-protection behaviors mediated by variables that people consider future consequences (Zhang et al., 2021). Moreover, it showed that health-related theories are clearly associated with mental health. A meta-analysis study suggested that the mental health level of young people who hold the ontological implicit belief (that personal attributes are fixed) is significantly lower than that of young people who hold the gradual implicit belief (that personal attributes are dynamic and malleable) (Schleider et al., 2015). This is because individuals who hold the entity theory believe that negative events are caused by stable internal causes, and they are more likely to have anxiety and depression because they feel that they cannot change negative events. On the contrary, when facing negative events, adolescents who hold the gradual change theory believe that the outcome of negative events can be improved through targeted efforts, and they tend to form a positive and optimistic attitude and exhibit lower anxiety levels. A recent study on the interaction of health messages on social media reinforces this view by demonstrating that lay theories of health play a moderating role, implying that when individuals hold incremental lay theories, they are more willing to interact with mindfulness information (Wang et al., 2023). We can reasonably infer that individuals who hold incremental lay theories of health have higher self-efficacy and tend to actively regulate health-related behaviors. Therefore, we predict that lay theories of health would moderate the association between health risk perception, health anxiety, and exercise intention (Hypothesis 3).

**Figure 1:**
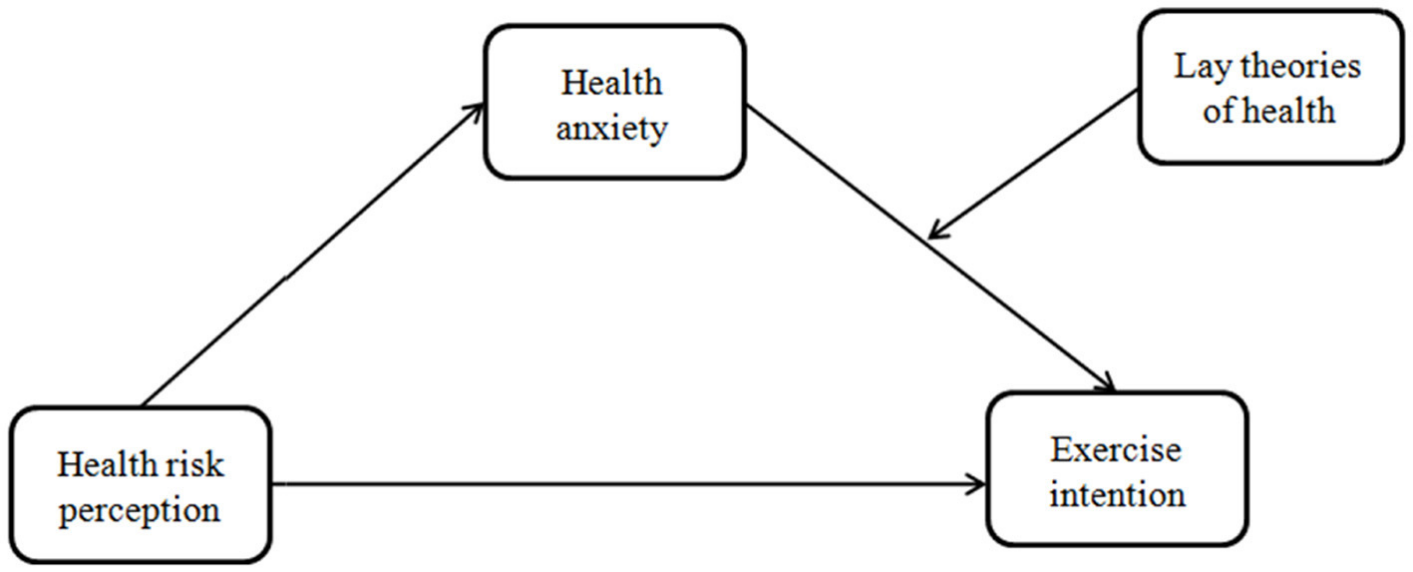
The proposed theoretical model.

From the above discussion, it is a clear that the underlying mechanism connecting health risk perception and exercise behaviors is not well understood. To sum up, in this study, we explored the antecedent variables and intrinsic mechanisms of physical exercise from the perspective of risk perception to provide a reference for improving college students' engagement in physical exercise. Based on previous studies and the theoretical framework, we aimed to investigate both the association between health risk perception and exercise intention of college students as well as the underlying mechanisms of this relationship, with a focus on the potential mediating effect of health anxiety. Furthermore, we postulated that the intermediate path is moderated by lay theories of health, implying that only individuals who hold the incremental theory have a greater willingness to exercise in the face of higher health risks and health anxiety (Figure 1).

## 2 Materials and methods

### 2.1 Participants

We recruited 826 college students from three universities in Henan province located in the central region of China from 10 January 2023 to 25 January 2023 using convenience sampling. The questionnaire was distributed to the participants. During the collection of questionnaire responses, 59 invalid questionnaires with regular answers and lack of data were excluded, with a recovery rate of 92.3%. A total of 767 valid questionnaires were obtained from 468 men and 299 women. The average age of the subjects was 19.614 ± 1.635 years. The completion time of each questionnaire was ~6–8 min. The test was conducted with the consent of the physical education (PE) teacher and participants themselves. A collective test was adopted, emphasizing the principles of anonymous and voluntary filling and data confidentiality.

### 2.2 Measurement

#### 2.2.1 Health risk perception

We adopted the Health Risk Perception Scale that consists of 10 items including two dimensions: perception of susceptibility and perception of severity (Zhou and Lin, 2020). The first six items describe the perception of susceptibility (e.g., “I feel that my health is vulnerable at present”) and the last four items describe the perception of severity (e.g., “Illness will have a bad influence on my social life”). The participants were required to respond to each item on a scale ranging from 1 (strongly disagree) to 7 (strongly agree). The average score was calculated as the level of health risk perception. A higher score represents a higher level of health risk perception. The results of confirmatory factor analysis confirmed that the scale had good validity [Model fitting index (χ^2^/*df*) = 4.261, root-mean-square error of approximation (RMSEA) = 0.064, comparative fit index (CFI) = 0.943, Standardized Root Mean Square Residual (SRMR) = 0.032]. The scale exhibited good reliability for the present study, with a Cronbach's α of 0.823.

#### 2.2.2 Health anxiety

Health anxiety was measured by a simplified version of the Chinese Health Anxiety Scale (Zhang et al., 2015). The scale consists of 18 items, which are rated on a scale from 0 (never) to 4 (always). A higher total score indicates a higher level of health anxiety. The result of confirmatory factor analysis indicates that the scale has good validity (χ2/*df* = 5.094, RMSEA = 0.070, CFI = 0.950, SRM*R* = 0.041). Cronbach's α of the health anxiety scale for this study was 0.841.

#### 2.2.3 Lay theories of health

The scale of the Lay Theories of Health scale comprises six items (Bunda and Busseri, 2019). Three items measure entity theory of health (e.g., “My health is a part of me that I cannot change very much”) and the remaining three items measure incremental theory of health (e.g., “I can change even my basic level of health considerably”). The participants rated the items on a 7-point Likert scale, ranging from 1 indicating strong agreement to 7 indicating strong disagreement. After reverse-scoring of the entity item ratings, the average score was calculated as the level of the incremental theory of health. A higher score represents a stronger incremental theory of health. The following confirmatory factor analysis result confirmed the validity of the scale: χ2/*df* = 4.174, RMSEA = 0.065, CFI = 0.932, SRM*R* = 0.037. Cronbach's alpha of the scale for the present study was 0.712.

#### 2.2.4 Exercise intention

The Chinese version of the Exercise Intention Scale, compiled by Ajzen (2002) and translated and revised by Fang (2012), was adopted to measure the exercise intention of the participants. The scale consists of three items (i.e., “I plan to exercise for at least 20 min at least three times a week, in the next 2 weeks,” “I intend to exercise for at least 20 min at least three times a week, in the next 2 weeks,” and “I hope to exercise for at least 20 min at least three times a week, in the next 2 weeks”). The participants rated each item on a 7-point-Likert scale, ranging from 1 indicating completely disagree to 7 indicating completely agree. A higher score indicates a stronger intention to exercise. The confirmatory factor analysis result confirmed good validity of the scale (χ2/*df* = 4.630, RMSEA = 0.076, CFI = 0.904, SRM*R* = 0.043). Cronbach's α of the exercise intention scale for this study was 0.920.

### 2.3 Data analysis

All statistical analyses were conducted using IBM SPSS Statistics for Windows. First, we conducted confirmatory factor analysis to detect a significant common method bias. Second, we derived the descriptive statistics for the main research variables and then conducted Pearson's correlation analysis to analyze the relationship between the variables. Third, the SPSS macro program compiled by Hayes in SPSS23.0 was used to verify the mediating role of health anxiety and the moderating role of lay theories of health.

## 3 Results

### 3.1 Common method bias test

We adopted Harman's single-factor method to test the common method bias. The results showed that the characteristic roots of 10 factors were >1, and the first factor could explain 13.823%, which was less than the standard critical value of 40%, thus indicating that there is no considerable common method bias in this study.

### 3.2 Descriptive statistics and correlation analysis

Table 1 shows the mean (M) and standard deviation (SD) for the main variables, as well as the correlation values. The Pearson correlation analysis test showed that health risk perception was significantly positively associated with health anxiety and exercise intention (*r* = 0.485, *p* < 0.01; *r* = 0.112, *p* < 0.05). Health risk perception had a significantly negative association with implicit health theory (*r* = −0.133, *p* < 0.01). Health anxiety was positively associated with exercise intention (*r* = 0.143, *p* < 0.01). There was a significantly positive association between health theory and exercise intention (*r* = 0.365, *p* < 0.01).

**Table 1:** Descriptive statistics and correlations between the main variables.

	***M±SD***	**1**	**2**	**3**	**4**
1. Health risk perception	16.70 ± 3.58	1			
2. Health anxiety	35.70 ± 4.47	0.485^**^	1		
3. Lay theories of health	4.34 ± 0.75	−0.133^**^	−0.054	1	
4. Exercise intention	15.66 ± 4.36	0.112^**^	0.143^**^	0.365^**^	1

^**^p < 0.01.

The results of the regression analysis for the mediating model are presented in Table 2, in which gender and age were included as control variables. Health risk perception was significantly associated with exercise intention (β = 0.110, *t* = 2.852, *p* < 0.01). Therefore, Hypothesis 1 is tenable. Health risk perception could significantly and positively predict health anxiety (β = 0.485, *t* = 15.193, *p* < 0.001). Health anxiety could positively predict exercise intention (β = 0.108, *t* = 2.565, *p* > 0.01). When both health anxiety and health anxiety were included in the regression equation as predictors of physical exercise behavioral intention, they could significantly and positively predict behavioral intentions toward physical exercise, and the mediating effect was significant. The bootstrap 95% confidence interval did not contain 0 [0.016, 0.099]. Hypothesis 2 is supported by the above data, indicating that health anxiety plays a mediating role between health risk perception and exercise intention. Furthermore, statistical results showed that the interaction between health anxiety and health theory had a significantly positive predictive effect on exercise intention in college students (β = 0.068, *t* = 2.067, *p* < 0.05). Therefore, Hypothesis 3 is supported.

**Table 2:** Regression model analysis.

**Dependent variables**	**Independent variables**	***R***	***R^2^***	***F***	**β**	***t***	**Bootstrap LLCI**	**Bootstrap ULCI**
Health anxiety	Gender	0.488	0.238	77.221^***^	0.015	0.173	−0.015	0.018
	Age				0.004	0.059	−0.08	0.008
	HRP				0.485	15.193^***^	0.422	0.548
Exercise intention	Gender	0.418	0.175	26.039^***^	−0.016	−0.185	−0.019	0.016
	Age				−0.042	−0.882	−0.013	0.005
	HRP				0.110	2.852^**^	0.034	0.186
	Health anxiety				0.108	2.565^**^	0.023	0.173
	Health theory				0.385	11.489^***^	0.320	0.451
	HA^*^ HT				0.068	2.067^*^	0.003	0.132

Gender: 0 = woman, 1 = man; ^*^p < 0.05, ^**^p < 0.01, ^***^p < 0.001. HRP, health risk perception; HA, health anxiety; HT, health theory; LLCI and ULCI are the lowest and highest values of the confidence interval, respectively.

**Figure 2:**
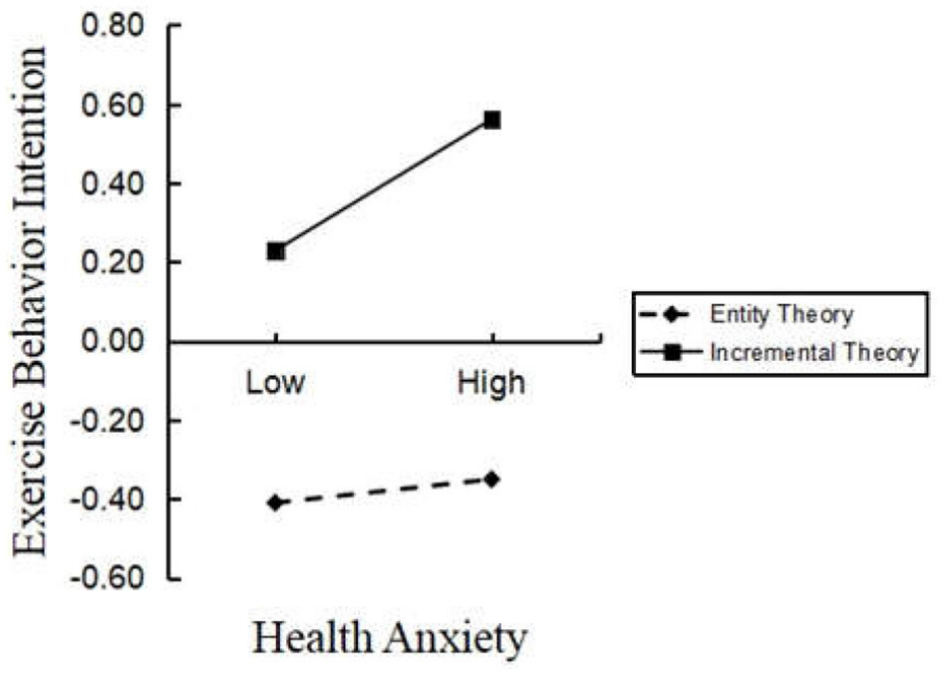
Interaction effects of health anxiety and lay theories of health on exercise intention.

To further portray the interaction, simple slope plots were obtained and β coefficients were calculated at −1SD and +1SD from the mean of health theory (Figure 2). The result of simple slope tests showed that, for college students with a lower score of health theory, the influence of health anxiety on exercise intention was statistically insignificant (β = 0.030, *t* = 0.605, *p* > 0.05; b_simple_ = 0.051, *p* > 0.05), with a 95% confidence interval of [−0.002, 0.104]. For college students with a higher score of health theory, the influence of health anxiety on exercise intention was positively and statistically significant (β = 0.166, *t* = 3.209, *p* < 0.05; b_simple_ = 0.139, *p* < 0.001), with a 95% confidence interval of [0.086, 0.192]. The results are shown in Table 3.

**Table 3:** Moderated mediation analysis.

**Dependent variable**	**Moderator**	**Effect**	**SE**	**Boot LLCI**	**ULCI**
Exercise intention	−1SD	0.014	0.029	−0.042	0.070
	M	0.048	0.019	0.011	0.086
	+1SD	0.081	0.025	0.035	0.134

## 4 Discussion

The present study investigated the relationship between health risk perception and exercise intention in Chinese college students as well as the underlying mechanism of this relationship. Our findings show that health risk perception can positively predict exercise intention with the mediating role of health anxiety. Furthermore, lay theories of health play a moderating role in the effect of health anxiety on exercise intention.

The results of this study show that there is a significant positive correlation between health risk perception and exercise intention, thus verifying Hypothesis 1. Many health behavior models propose that risk perception is a key contributor to people's willingness to undertake behavioral changes (Yildirim et al., 2020; Bonilla-Asalde et al., 2023). Risk perception plays an important role in human self-protection and social behavior. In this regard, it is important to study individual perceptions of health risks because perceptions toward health risks can primarily affect initiating or maintaining positive health behaviors or avoiding negative ones. When the level of perceived risks is high, people are highly sensitive to all types of risk information and exhibit a high inclination for health preventive behaviors. However, there is insufficient literature on the effect of risk perception on exercise behavior. Previous studies have shown that the risk perception of COVID-19 has a significant and positive impact on the persistence in prevention and exercise behaviors. There is also supporting evidence that the general population's appropriate coping behavior is positively associated with their physical exercise engagement during the COVID-19 epidemic (Luo et al., 2020). It is not surprising that health risk perception can positively predict exercise intention. Regular physical exercise is a core element and indicator of a healthy lifestyle, which can bring significant physical and mental benefits to individuals. Exercise may also protect against diseases by enhancing the body's immune system. Therefore, when college students perceive a high health risk, they tend to make certain protective responses to reduce the adverse situation caused by stressors, such as engaging in strengthening exercise to improve resistibility. For example, the proportion of people participating in sports activities increased significantly during the COVID-19 pandemic.

The public's emotional experiences and risk perceptions have always been one of the most important topics of research during public health events. The emergence of the COVID-19 pandemic resulted in major changes in daily life and economic conditions while heightening physical and mental health problems, including health anxiety. This study found that health anxiety plays a mediating role between health risk perception and exercise intention, supporting Hypothesis 2. In other words, a high level of health risk perception in individuals has an impact on their physical exercise intention through health anxiety. Many studies have found that, in the context of a public health event, the general population may develop negative emotions such as fear and anxiety, which in turn increase the implementation of preventive behaviors (Kim et al., 2015; Liu et al., 2021). Intuitively, anxiety occurs when an individual perceives that his or her health is under threat. The cognitive behavioral model proposes that individuals often engage in distinctive behaviors in an effort to cope with or reduce health anxiety. Indeed, physical activity is widely recognized as a modifiable factor that offers protection against health conditions (Weinstein et al., 2008). A recent study suggested that anxiety may contribute to individuals' engagement in compulsive exercise (Palermo and Rancourt, 2023). Therefore, individuals may engage in physical exercise in an attempt to assuage health anxiety by promoting health. In fact, there is evidence to support that individuals with high levels of health anxiety who treat exercise as a way of coping with anxiety have a greater desire to exercise (Pugh and Hadjistavropoulos, 2011; Back et al., 2021). Based on the above analysis, it is logical to infer that health anxiety plays a mediating role between health risk perception and exercise intention.

Although health risk perception of college students may be significantly associated with exercise intention through the mediating role of health anxiety, this relationship is not stable or unchanging. Therefore, the present study also explored potential moderating variables that may influence the relationship between health risk perception, health anxiety, and exercise intention. By exploring the lay theories of health adopted by college students, we can implement effective interventions to alleviate their anxiety, help them cope with frustrating events, and improve their exercise intention. Consistent with our hypothesis, lay theories of health moderate the relationship between health anxiety and exercise intention. Specifically, individuals holding the incremental health theory exhibit a stronger positive prediction effect of health anxiety on exercise behavior intention than those holding the entity health theory. As demonstrated above, health risk perception may lead to continuous negative emotions like health anxiety. However, individuals holding entity theories of mental health believe that their health is fixed, preventing them from making efforts toward changing it. Consequently, this belief makes individuals reluctant to consider physical exercise as a measure to cope with health anxiety. Therefore, health anxiety in individuals holding entity health theories has no significant effect on the prediction of exercise intention. Conversely, individuals holding incremental theories of health believe that health is dynamic, malleable, and developable and it can change their health through their own efforts. Similarly, research suggests that people with incremental theories would adopt more active coping styles, focusing their energy on responding to anxiety (Dweck, 1999). Correlational and experimental findings indicated that individuals holding incremental (vs. entity) lay theories are characterized by more effective goal pursuit, emotion regulation, and motivation (Burnette et al., 2013; Yeager et al., 2014). Such results have been attributed to various processes, including incremental lay theorists' more effective strategies for coping with negative feedback and their greater willingness to invest time and effort in improving their condition. It is not unreasonable to infer from the results of this study that individuals holding the incremental health theory are more inclined to show greater exercise intention when faced with health anxiety caused by health risk perception. These findings are consistent with previous research conclusion that, compared with individuals holding entity theorists, those holding more incremental theories of health have stronger intentions to pursue more health-promoting behaviors. Thus, people with a higher level of incremental health theory believe that they can improve their health status through their own efforts and have stronger intentions to engage in healthy lifestyles such as exercising and healthy eating (Zhang and Kou, 2022). These findings suggest that individuals' lay theories of health (changeable or fixed) can affect exercise intention. In particular, individuals who think that their health can be changed to a large extent believe that their health will improve greatly over time through a healthy lifestyle.

## 5 Implications and future research prospect

This study offers multiple theoretical implications. First, it reveals the association between health risk perception, emotions, and behaviors in the post-pandemic era. Second, it enriches an understanding of the mechanism underlying the association between health risk perception and exercise intention by demonstrating the mediating effect of health anxiety. Finally, the study results show that the lay theories of health play a moderating role in the effect of health anxiety on exercise intention. This study also provides key insights from a practical perspective, which will help in the development of practical applications. Increased risk perception will motivate people to adopt coping behaviors. For example, if individuals are encouraged to enhance their physical fitness based on individual efforts when they perceive health risks, such propaganda may lead them to adopt a stronger incremental lay theory, which will enhance their motivation to implement health-related behaviors.

The following limitations of the study are recognized and will be addressed in future research. The data were obtained from an undergraduate student sample with a limited age range. A replication of this study would be useful using community samples with a more representative average age. Moreover, health risk perceptions and ways of coping with health anxiety differ among different populations, especially among the elderly or individuals with chronic diseases such as cardiovascular and cerebrovascular diseases. People's perceptions on health risks also depend on their individual differences, including personality traits, gender, culture, and prior experience (Menon et al., 2006; Kim, 2018; Dryhurst et al., 2020). Therefore, future research should focus on the different age and culture groups. The causal hypotheses could not be tested owing to our use of a cross-sectional design. Future work should include longitudinal, experimental, or intervention research methods to determine the causal nature of the relationship. Finally, other mechanisms should be examined to clarify the relationship between health risk perception and exercise intention.

## 6 Conclusion

This study presented the role of health anxiety and lay theories of health in the relationship between health risk perception and exercise intention in college students. Health risk perception positively affects exercise intention by aggravating health anxiety among individuals, and lay theories of health can moderate the effects of health anxiety on exercise intention. These findings have both theoretical and practical implications. Theoretically, this study expanded the research on the positive outcomes of health risk perception and the factors influencing exercise intention. In particular, it revealed the mechanism underlying the relationship between health risk perception and exercise intention by integrating the emotional and cognitive factors.

## Data availability statement

The original contributions presented in the study are included in the article/supplementary material, further inquiries can be directed to the corresponding author.

## Ethics statement

The studies involving humans were approved by the School of Education, Zhengzhou University. The studies were conducted in accordance with the local legislation and institutional requirements. The participants provided their written informed consent to participate in this study.

## Author contributions

KW: Conceptualization, Methodology, Writing – original draft. CL: Investigation, Methodology, Writing – review & editing. XY: Data curation, Formal analysis, Methodology, Writing – review & editing. YW: Conceptualization, Methodology, Writing – original draft.
